# Study on the Hyperglycemic Effect of GLP-1 in *Spinibarbus denticulatus* by Oral Administration and Intraperitoneal Injection Methods

**DOI:** 10.1155/2023/9969406

**Published:** 2023-04-03

**Authors:** Wei Luo, Luojia Li, Yue Zhang, Zhou Xu, Yinlin Xiong, Zhonggang Guo, Ning Zhang, Yibo Zhang, Pengyu Chen, Yan Wang, Zongjun Du

**Affiliations:** ^1^College of Animal Science and Technology, Sichuan Agricultural University, Wenjiang, Chengdu, Sichuan, China; ^2^Mianyang Academy of Agricultural Sciences, Mianyang, Sichuan, China; ^3^The Original Stock Farm of Leiocassis longirostris of Sichuan Province, Chongzhou, Sichuan, China; ^4^Agricultural and Rural Bureau of Chongzhou, Chongzhou, Sichuan, China

## Abstract

Glucagon-like peptide-1 (GLP-1), one of the expression products of the proglucagon (pg) gene, is an incretin mainly secreted by the gastrointestinal system. In mammals, GLP-1 has hypoglycemic and food-inhibiting effects; while in some fish species, it has been confirmed to increase blood glucose by promoting gluconeogenesis and stimulating glycogenolysis. In order to more deeply understand the role of GLP-1 in the process of glycometabolism in herbivorous fish, the pg gene was cloned from *Spinibarbus denticulatus* to obtain its sequence characteristics, and the changes in blood glucose level and pg gene expression in *S. denticulatus* were further explored by feeding with three kinds of carbohydrates and intraperitoneal injection of GLP-1. Basal and temporal blood glucose levels and pg gene expression of *S. denticulatus* (91.68 ± 10.79 g) were measured at 0, 1, 3, 5, 7, and 12 h after oral administration (*n* = 4). Then, the changes of blood glucose levels and pg and glucokinase (gk) gene expressions of *S. denticulatus* (94.29 ± 10.82 g) were determined at 0, 30, 60, and 120 min after intraperitoneal injection (*n* = 4). It was shown that polysaccharides could induce the upregulation of pg gene expression faster than monosaccharides and stimulate the secretion of GLP-1 in the intestine. Intraperitoneal injection of GLP-1 peptide rapidly raised blood glucose levels, and pg gene expression in the anterior intestine, whole brain, and hepatopancreas decreased continuously after 30 minutes. These results showed that *S. denticulatus* might inhibit the excessive accumulation of blood glucose by reducing the expression of the pg gene and increasing the expression of gk gene in a short time. It was speculated that GLP-1 of *S. denticulatus* might have a “gut-brain-liver” pathway similar to mammals in glycemia regulation. Therefore, this study provided a novel perspective for explaining the functional differences of GLP-1 in herbivorous fish and mammals.

## 1. Introduction

Glucagon is an important factor in the regulation of blood glucose homeostasis in animals, which can promote the process of gluconeogenesis and inhibit the synthesis of hepatic glycogen to increase blood glucose levels. The expression product of proglucagon (pg) is glucagon-like peptide (GLP), which has two forms in mammals: GLP-1 and GLP-2 [1, 2]. All fish pg genes have GLP-1 expression products, while GLP-2 only exists in some fish, and there are few literature reports. The functions of GLP such as the control of food intake and glycemia regulation are mainly completed by GLP-1 [3, 4].

In mammals, GLP-1 is mainly secreted by L cells in the terminal ileum, colon, and rectum [5]. It is an incretin that promotes insulin secretion and inhibits the release of glucagon. GLP-1 has a hypoglycemic effect by converting carbohydrates into noncarbohydrates, such as fat, for energy storage [6]. Additionally, mammalian brain neurons can produce GLP-1 to regulate ingestion behaviors [7, 8]. GLP-1 receptors reduce food intake by inhibiting adenosine 5′-monophosphate-activated protein kinase (AMPK) and activating the mammalian target of rapamycin (mTOR) [9]. Current studies on carnivorous fish such as rainbow trout (*Oncorhynchus mykiss*) [10], copper rockfish (*Sebastes caurinus*) [11], and omnivorous channel catfish (*Ictalurus punctatus*) [12] have all shown that GLP-1 has a hyperglycemic effect similar to glucagon. GLP-1 in fish has a food-inhibiting effect similar to that in mammals, while the function in blood glucose regulation is opposite to that of mammals. GLP-1 in fish promotes gluconeogenesis and hepatic glycogenolysis to increase blood glucose levels but has no effect on insulin secretion [13, 14].

The glucose turnover rate of fish is lower than that of mammals; it has the characteristics of “hyperglycemic intolerance” and “hypoglycemic tolerance” [15]. Compared with mammals, the activity of carbohydrate digestive enzymes in fish is lower, and the glucose metabolic enzymes and hormone secretion are not synchronized with food intake [16]. Fish have slow insulin secretion and a lack of insulin receptors [17], their glycolytic enzyme activity is inhibited after ingestion of dietary carbohydrates, and the gluconeogenesis potency is enhanced [18]. This indicates a persistent hyperglycemia phenomenon, which is similar to diabetes mellitus type 2 (T2DM) symptoms in mammals [19]. In the glucose tolerance tests, it was found that blood glucose in mammals generally returned to normal levels within 1.5-3 h, while it took 5-12 h for omnivorous fish and 12-36 h for carnivorous fish [20, 21]. Compared with mammals, fish do not have a relatively integrated balance regulatory system or a complex neural network to regulate glucose metabolism [22, 23]. When studying the mechanism of glycometabolism in fish, the differences in glucose metabolism between fish and mammals should be considered from the perspectives of species evolution, different living environments, and the complex physiological functions [24]. Many studies have shown that GLP-1 has significantly different physiological functions across different species, while the mechanism of blood glucose regulation mediated by GLP-1 and key gene expression rules in herbivorous fish are still unclear. The differences in glucose tolerance and glycometabolism regulation mechanism in fish and mammals can be further explored through GLP-1.

*Spinibarbus denticulatus* is an economic species with a development value in the Pearl River, China. The fish is characterized by large size, fast growth, and tender meat quality. More and more attention has been paid to its introduction, domestication, large-scale culture, and artificial propagation. The adult fish is herbivorous, which is a classical model for studying the regulation of glycometabolism [25]. Therefore, *S. denticulatus* was used as the model to explore the involvement of the pg gene in the process of blood glucose regulation in herbivorous fish. Firstly, the pg gene was cloned, and tissue distribution was analyzed. The changes in blood glucose level and relative gene expressions in *S. denticulatus* were further explored by oral administration of three kinds of carbohydrates and intraperitoneal injection of GLP-1 peptide. The data will provide a novel perspective for understanding the process of blood glucose regulation mediated by GLP-1 in herbivorous fish.

## 2. Materials and Methods

### 2.1. Ethics Statement

Our study did not involve endangered or protected species. All the fish were cultured in ponds at the Sichuan Agricultural University laboratory and fed with artificial feeds. Fish treatments were carried out seriously according to the Guide for the Care and Use of Laboratory Animals of the National Veterinary and Quarantine Service. The animal study was reviewed and approved by the Science and Technology Bureau of China. The fish was appropriately anesthetized with 30 mg/L MS-222.

### 2.2. Animal, Oral Administration, and Intraperitoneal Injection

*S. denticulatus* were purchased from Pengshi's special aquatic seedling farm in Sichuan Province, China. These fish were maintained in a circular laboratory pool (radius × height: 47.5 cm × 85 cm, water volume: 400 L) for 2 weeks, aquatic water was aerated for 24 h in advance, and 1/3 of the water was changed every day. Continuous oxygenation was performed to maintain dissolved oxygen (DO) at 7.45 ± 0.30 mg/L. The water temperature was 22 ± 0.5°C, pH was 7.2 ± 0.3, and photoperiod was 12 L: 12 D. The fish was fed twice (9:00 and 16:00) every day with a commercial universal feed for freshwater fishes (Tongwei Company, Chengdu, China; approximate composition: crude protein ≥ 30.0%, crude fiber ≤ 12.0%, crude lipid ≥ 5.0%, crude ash ≤ 15.0%, moisture ≤ 12.5%), and the feeding ratio was approximately 3%. Healthy fish (*n* = 64) with an average weight of 91.68 ± 10.79 g were selected. After fasting for 48 h, fish were lightly anesthetized with MS-222 (30 mg/L), and analytical glucose, maltose, or soluble starch was administered orally at 1.67 g/kg body weight (BW). Healthy fish (*n* = 28) with an average body weight of 94.29 ± 10.82 g were additionally selected. After fasting for 48 h, these fish were lightly anesthetized with MS-222 and intraperitoneal injected with normal saline (1 *μ*L/g BW, 0.65%), DEPC water (1 *μ*L/g BW) or GLP-1 peptide at concentrations of 500 *μ*g/kg BW (500 ng/*μ*L), 750 *μ*g/kg BW (750 ng/*μ*L), 1000 *μ*g/kg BW (1000 ng/*μ*L), 1250 *μ*g/kg BW (1250 ng/*μ*L), or 1500 *μ*g/kg BW (1500 ng/*μ*L). The range of dosages of GLP-1 was set according to our preexperiment and the experiment designed by Polaof et al. [10]; then, the optimal concentration of GLP-1 was subsequently chosen. GLP-1 polypeptide dry powder with a purity of 95% was synthesized in Shanghai Botai Biotechnology Co., Ltd. The N'-C' amino acid sequence was HAEGTYTSDVSSCLQDQAAQNFVAWLKSGQP. DEPC water was used to configure the GLP-1 solution.

### 2.3. pg Gene Cloning

After fasting for 24 h, 2 hepatopancreas samples and 2 middle intestine samples were collected and stored in a -80°C ultralow temperature freezer. The hepatopancreas and middle intestine tissues were fully ground with liquid nitrogen in a sterilized mortar. RNA was extracted according to the manufacturer's instructions (total RNA extraction kit, Dalian Takara (RNAiso Plus reagent)). RNA quality was detected by the ultralow volume spectrometer and nondenaturing agarose gel electrophoresis. RNA samples were free of protein and DNA contamination. After electrophoresis on 1.0% agarose gel, clear bands could be seen (Figure S1). The brand distribution of the second sample was more uniform, and the degradation was little, indicating that the integrity of the second sample was better than that of the first sample. RNA extracted from the second midgut and hepatopancreas sample was selected as a template for cloning the pg gene. cDNA preparation was performed according to the manufacturer's instructions (Reverse Transcription Kit, TaKaRa company, RR037A).

According to the sequence of the homology region of the pg gene of *Carassius auratus* (GB: U65528.1) and *Schizothorax prenanti* (GB: KM232679.1), the *S. denticulatus* pg gene-specific primer pg-1F, pg-1R, pg-2F, pg-2R was designed to clone the core fragment (Table S1). According to the obtained *S. denticulatus* pg gene core sequence and specific primer design requirements for RACE technology, downstream primers pg-gsp1 and UPM (UPM was the universal primer in the kit; its specific sequence was shown in Table S1) were designed for the amplification of the 5′-end sequence of pg gene cDNA. The upstream primer pg-gsp1-L and UPM were used for the first PCR amplification of the 3′-end sequence, and pg-gsp1-S and UPM used the first PCR product as a template (diluted 50 times) for the second PCR amplification. The 5′-end and 3′-end sequences were compared for correctness by BLAST (https://blastpremier.com); sequence splicing was performed by DNAMAN 9.0 to obtain the full-length cDNA sequence. The obtained pg gene of *S. denticulatus* full-length cDNA sequence was submitted to the NCBI database.

### 2.4. Bioinformatics Analysis of Amino Acid Sequence of pg Gene

The amino acid sequence of the protein encoded by the *S. denticulatus* pg gene was deduced using DNAStar software, and the phosphorylation site of the pg amino acid sequence was predicted by NetPhos 3.1 software. The spatial structure of the *S.denticulatus* pg protein was predicted by SWISS-MODEL software. Clustal X (1.83) was used to calculate the homology of the *S. denticulatus* pg amino acid sequence with other species such as *Sinocyclocheilus anshuiensis*, *Sinocyclocheilus rhinocerous*, *Cyprinus carpio*, *S. prenanti,* and multiple comparisons performed. The phylogenetic tree was constructed using MEGA 5.0 software, and the reliability of the estimated tree was evaluated by the bootstrap method with 1000 pseudoreplications.

### 2.5. Determination of Blood Glucose and Hepatic Glycogen Levels after Different Treatments

Blood glucose level was determined by the glucose oxidase-peroxidase (GOD-POD) method, and specific operation steps were carried out according to the blood glucose determination kit (Shanghai Rongsheng Biopharmaceutical Co., Ltd.). For measuring the basal blood glucose level, after the experimental fish were maintained for 2 weeks (feeding methods were shown in 2.1) and anesthetized with MS-222, blood samples (*n* = 9; about 1 mL per fish) were collected from tail veins at 48 h and 288 h after fasting, respectively.

The drenched fish (*n* = 64) were anesthetized with MS-222, and we took samples (*n* = 4) at 0, 1, 3, 5, 7, and 12 h after drenching with three different kinds of carbohydrates. Blood was collected from the tail vein, and serum was prepared by centrifugation at 4°C and 3000 r/min for 10 min. The supernatant was transferred to a centrifuge tube and stored at 4°C until use for blood glucose determination. After blood collection, the hepatopancreas were quickly collected and stored in liquid nitrogen for hepatic glycogen determination. Glycogen were determined according to the kit (Nanjing Jiancheng Institute of Biology); the determination principle was that glycogen can be dehydrated to form a furaldehyde under the action of concentrated sulfuric acid, and then react with anthrone to form a blue compound. The injected fish (*n* = 28) were anesthetized, and we took blood samples (*n* = 4) from the tail veins at 0, 30, 60, and 120 min after intraperitoneal injection of normal saline or GLP-1 to determine the blood glucose level.

### 2.6. Determination of Basal pg Gene Expression in Adult *S. denticulatus*

After the fish were anesthetized with MS-222, samples were taken immediately. Blood (1 mL) was taken from the tail vein, and 200 *μ*L was quickly transferred to a centrifuge tube with 1 mL of RNAiso Plus reagent and stored at 4°C. After blood collection, 18 tissues were collected: the head kidney, middle kidney, hepatopancreas, spleen, ovary, testis, anterior intestine, middle intestine, posterior intestine, heart, gill, brain, eyes, red muscle, white muscle, swim bladder, skin, and fin rays, and stored at -80°C for total RNA extraction.

With reference to the obtained full cDNA sequence of the *S. denticulatus* pg gene, a pair of quantitative primers pg-yg-F and pg-yg-R was designed across at least one intron, referring to a pair of internal reference gene primers *β*-ActinF1, *β*-ActinR1 designed by Zhu et al. [25]. Specific primers (EF-1*α*F1 and EF-1*α*R1) were designed according to the homology region sequence of elongation factor-1 alpha (EF-1*α*) gene of grass carp, common carp, and crucian carp for another internal reference gene EF-1*α* core fragment cloning. Then, a pair of reference gene quantitative primers EF-1*α*-yg-F and EF-1*α*-yg-R was designed according to the core fragment. All primers were sent to Chengdu Qingkezixi Co., Ltd. for synthesis (Table S2). Real-time fluorescence quantitative reaction of the pg gene was carried out on a Bio-Rad CFX96 quantitative PCR instrument according to the instructions of fluorescence quantitative (Dalian Bao Bio Co., Ltd. RR820A).

### 2.7. Determination of Temporal Changes of Gene Expression after Different Treatments

The basis for the grouping of fish was the same as when blood glucose level was measured, samples (*n* = 4) were taken at 0, 1, 3, 5, 7, and 12 h after drenching with three kinds of carbohydrates. After blood collection, the hepatopancreas, whole brain, and anterior intestine tissues were quickly collected and stored in liquid nitrogen for real-time PCR to examine the effects of GLP-1 on gene expressions of pg and glucokinase (gk). gk is the rate-limiting enzyme in glycolysis. After intraperitoneal injection of 1000 g/kg BW GLP-1 (the optimal dosage we chose), the hepatopancreas, whole brain, and anterior intestine samples were taken at 0, 30, 60, and 120 min. The amplification underwent the same processes as the determination of basal pg gene expression.

### 2.8. Statistical Analysis

Quantitative data were expressed as the means ± standard, and all statistics were analyzed by SPSS Statistics 20.0 (SPSS, Chicago, IL, USA). Significant differences were estimated by two-way analysis of variance (ANOVA) followed by Duncan's multiple range tests for the analysis of blood glucose level and relative gene expressions under different treatments.

## 3. Results

### 3.1. pg Gene Sequence Alignment and Phylogenetic Analysis

**Figure 1:**
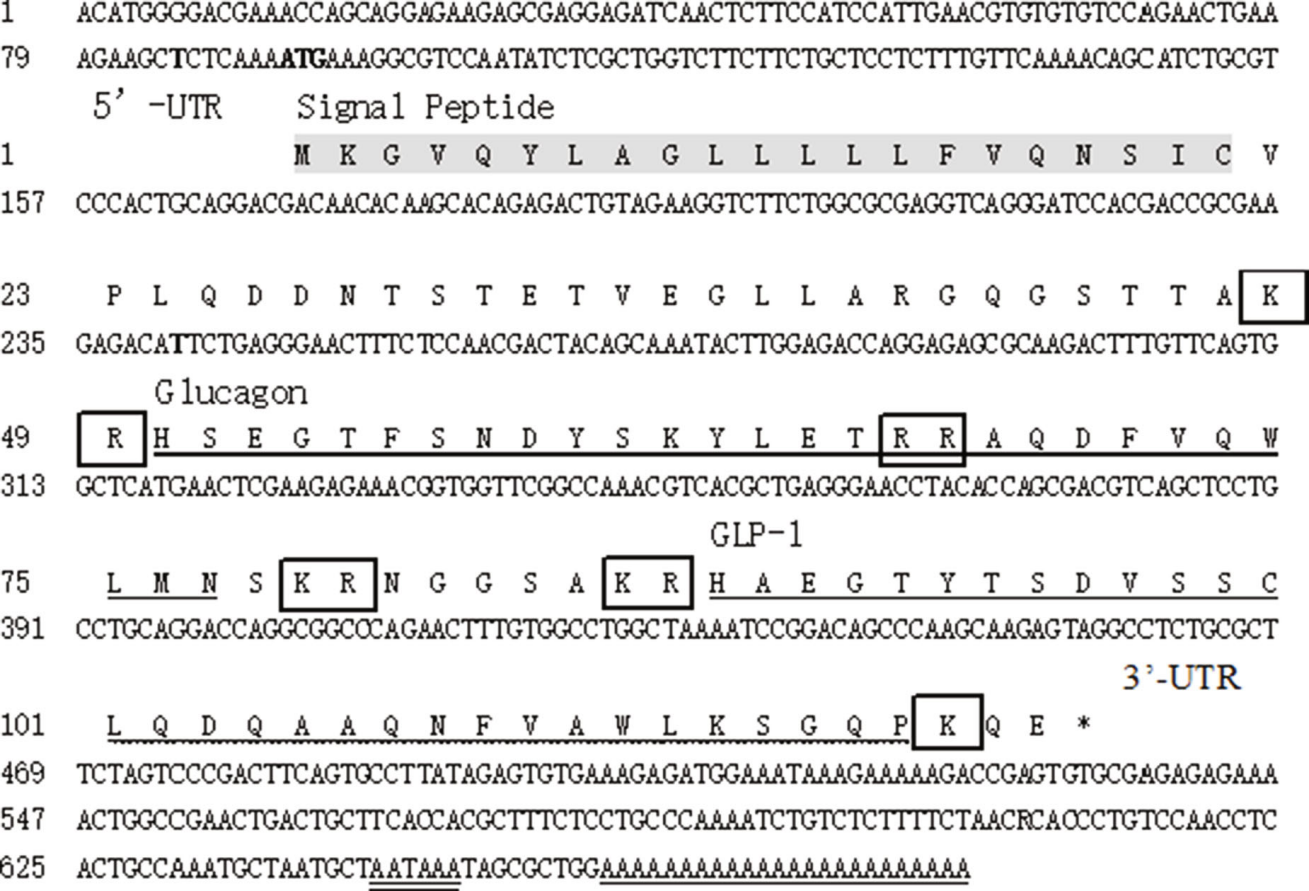
The pg complete cDNA sequences and predicted amino acids of *S. denticulatus.* The start codon is boldfaced. The stop codon is indicated by the “^∗^”. The gray highlighted characters in proper order indicate the signal peptide. Glucagon is underlined with “⎯”, and GLP-1 is underlined with “﹏”. Putative proteolytic cleavage sites are boxed, the add the tail signal of AATAAA is underlined with “═”, and the poly(A) is underlined with “_”.

The total length of the pg gene was 682 bp, with 91 bp 5′-UTR and 225 bp 3′-UTR; the opening reading frame (ORF) was 366 bp (Figure 1). The pg gene encoded a protein with 121 amino acids and a molecular weight of 13.41 kDa and *pI* = 7.78. There were 13 positively charged amino acid residues (Arg and Lys) and 12 negatively charged amino acid residues (Asp and Glu), suggesting that the protein encoded by the pg gene of *S. denticulatus* might be positively charged. It incorporated a proteolytic cleavage site, signal peptide, glucagon, and glucagon like peptide-1 (GLP-1) (Figure S2). The stop codon appeared immediately after the GLP-1 spliceosome, and the GLP-2 spliceosome did not appear in the sequence. SWISS-MODEL was used to obtain the three-dimensional spatial conformation of the *S. denticulatus* pg protein (https://swissmodel.expasy.org/), which was a typical secreted protein (Figure S3).

Clustal X (1.83) was used to calculate the homology of the amino acid sequence of the *S. denticulatus* pg with other species and was shown to be 47.5%-96.7%. The homology with the other cyprinids *S*. *anshuiensis*, *S. rhinocerous*, *C. carpio*, and *S. prenanti* was 96.7%, 96.7%, 95.0%, and 95.0%, respectively. Compared with other teleosts and terrestrial vertebrates, the homology was relatively low (Table S3& Figure S4). The results of the phylogenetic tree showed that *S. denticulatus* firstly formed a branch with *S. anshuiensis*, *S. rhinocerous*, *C. carpio*, and *S. prenanti*, indicating that the *S. denticulatus* pg gene had the closest homology to these cyprinid fishes. The cyprinid fish then merged into a branch with other teleost fish. *Ambystoma tigrinum* formed a branch with *Xenopus laevis*. Mammalian *Mus musculus* formed a branch with *Sus scrofa* (Figure S5).

### 3.2. Basal Expression of pg Gene in Adult *S. denticulatus* Tissues

**Figure 2:**
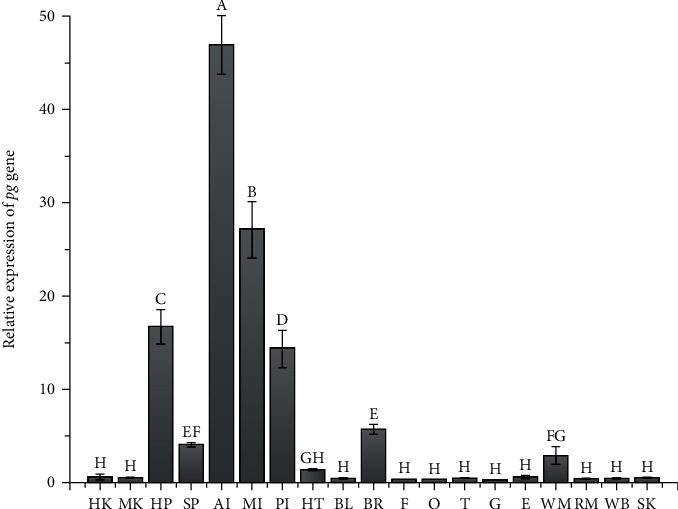
Tissue distribution of pg gene in adult *S. denticulatus* under normal condition. HK: head kidney; MK: middle kidney; HP: hepatopancreas; SP: spleen; AI: anterior intestine; MI: middle intestine; PI: posterior intestine; HT: heart; BL: swimming bladder; BR: brain; F: fin; O: ovary; T: testis; G: gill; E: eye; WM: white muscle; RM: red muscle; WB: whole blood; SK: skin. Different uppercase letters on bars indicate significant differences (*P* < 0.05).

There were significant differences in the expression levels of the pg gene in 19 tissues of adult *S. denticulatus* (*P* < 0.05; Figure 2). The expression level of the pg gene was the highest in the anterior intestine, which was significantly higher than other tissues (*P* < 0.05). For comparative analysis, the relative expression of pg gene in the heart was 1, the anterior intestine was 46.60, the hepatopancreas was 16.38, the middle intestine was 26.75, the posterior intestine was 14.04, the brain was 5.40, the white muscle was 2.61, the spleen was 3.77, and the remaining tissues were less than 1.

### 3.3. Temporal Changes of Blood Glucose and Hepatic Glycogen Levels after Oral Administration of Carbohydrates

After fasting for 48 h and 288 h, the basal blood glucose level of *S. denticulatus* remained stable, with no significant difference between the two groups (*P* < 0.05), but there was still a certain fluctuation (Table S4). The blood glucose value (mean ± S.D.) after 48 h of fasting was 4.07 ± 0.66 mmol/L, and the blood glucose value (mean ± S.D.) after 288 h of fasting was 3.73 ± 0.67 mmol/L.

**Figure 3:**
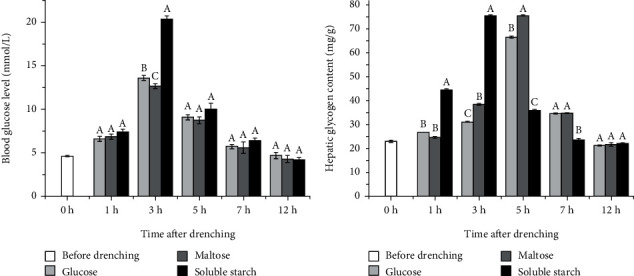
The effects of oral administration with different carbohydrates on (a) blood glucose and (b) hepatic glycogen level of *S. denticulatus*. At the same time, different uppercase letters indicate the values with significant differences (*P* < 0.05).

After drenching with three different kinds of carbohydrates, the blood glucose content of *S. denticulatus* showed a trend of firstly increasing and then decreasing (Figure 3(a)). The blood glucose level of each group peaked at 3 h after drenching and, finally, recovered to normal levels after 12 h. After 3 h of drenching, the blood glucose level in the soluble starch group was significantly higher than that in the other two groups (*P* < 0.05). After 5 h and 7 h of drenching, the blood glucose content in the soluble starch group was slightly higher than that in the other two groups.

After feeding with different kinds of carbohydrates, the level of hepatic glycogen in the three groups showed a trend of firstly increasing and then decreasing (Figure 3(b)). The hepatic glycogen level in each group peaked at 3-5 h after oral administration and returned to normal level after 12 h. At 1 h and 3 h after administration, the level of hepatic glycogen in the soluble starch group was significantly higher than that in the other two groups (*P* < 0.05). At 5 h and 7 h after administration, the hepatic glycogen content of the soluble starch group decreased, which was significantly lower than that in the other two groups (*P* < 0.05). These results indicated that polysaccharides and oligosaccharides affected blood glucose and hepatic glycogen levels with time differences.

### 3.4. Temporal Expression of pg Gene after Oral Administration of Carbohydrates

**Figure 4:**
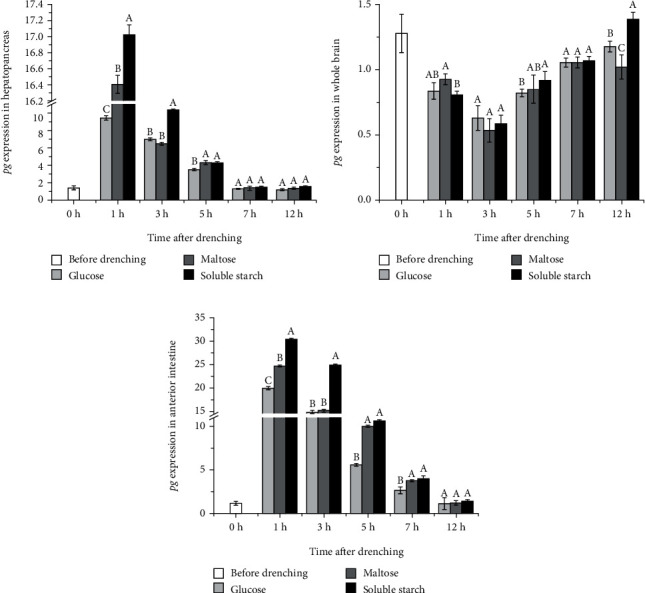
The effects of oral administration of different carbohydrates on pg gene expression in the hepatopancreas, whole brain, and anterior intestine tissues. Different uppercase letters indicate the values with significant differences (*P* < 0.05).

The expression level of the pg gene in the hepatopancreas and anterior intestine increased in a short time and then decreased. At 1 h and 3 h after drenching, the pg gene expression in the soluble starch group was significantly higher than that in the other two groups (*P* < 0.05; Figures 4(a) and 4(c)). However, the expression of the pg gene in the whole brain decreased at first and then increased. At 3 h after drenching, pg gene expression in the glucose group was significantly higher than that in the other two groups (*P* < 0.05), and pg gene expression in the soluble starch group was significantly higher than that in the maltose group (*P* < 0.05). At 5 h, the expression of the pg gene in the soluble starch group was significantly higher than that in the other two groups (*P* < 0.05; Figure 4(b)). After 12 h, the expression of the pg gene in the three groups roughly returned to the levels before administration.

### 3.5. Effects of Intraperitoneal Injection of GLP-1 on Blood Glucose Levels

**Figure 5:**
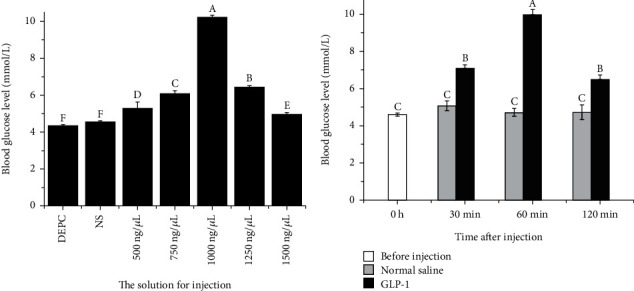
Effects of intraperitoneal injection of GLP-1 on blood glucose levels of *S. denticulatus*. (a) The effects of different concentrations of DEPC water, normal saline (NS), and GLP-1 injection on blood glucose. (b) The effects of GLP-1 injection on blood glucose levels at different time points. Different uppercase letters indicate the values with significant differences (*P* < 0.05).

After intraperitoneal injection of five different concentrations of GLP-1 for 1 h, the blood glucose level of *S. denticulatus* was significantly altered (Figure 5(a)). After intraperitoneal injection of 1000 g/kg BW GLP-1, blood glucose level increased to 10.25 mmol/L and was significantly higher than that in other groups (*P* < 0.05). Intraperitoneal injection of DEPC water and normal saline showed no significant differences in blood glucose concentration (*P* > 0.05). Therefore, the concentration of 1000 g/kg BW was optimal for intraperitoneal injection of GLP-1.

GLP-1 (1000 g/kg BW) was injected intraperitoneally into *S. denticulatus*. Blood glucose levels first increased, reaching the highest value at 60 min, and then decreased (Figure 5(b)). The GLP-1 group always had higher blood glucose levels than the normal saline group at the same time point, suggesting that GLP-1 could rapidly increase blood glucose in *S. denticulatus.*

### 3.6. Temporal Expression of pg and gk Genes after Intraperitoneal Injection of GLP-1

**Figure 6:**
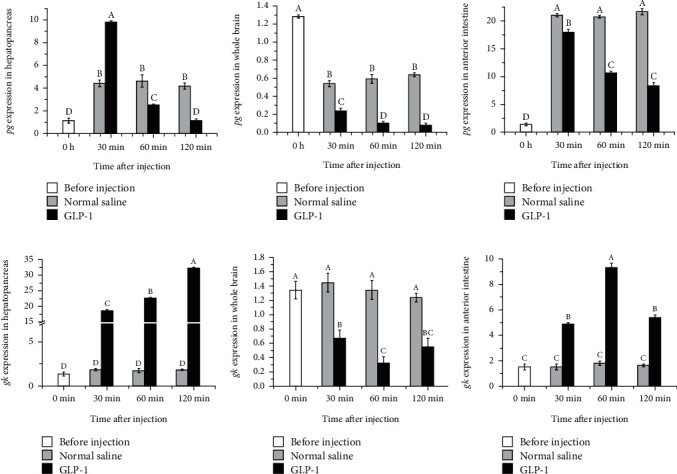
The effects of GLP-1 injection on pg and gk gene expressions in the hepatopancreas, whole brain, and anterior intestine tissues at different time points. Different uppercase letters indicate the values with significant differences (*P* < 0.05).

After injection of GLP-1, the expression of the pg gene in the hepatopancreas and anterior intestine increased briefly within 30 minutes. After 30 minutes, the expression levels of the pg gene in different tissues trended downwards. In the hepatopancreas, the expression of the pg gene in the GLP-1 group was significantly higher than that in the normal saline group at 30 min (*P* < 0.05), and then significantly lower than that in the normal saline group at 60 min and 120 min (*P* < 0.05; Figure 6(a)). In the whole brain, the expression of the pg gene in the GLP-1 group and normal saline group was significantly lower at 30-120 min than that before injection (*P* < 0.05), and the expression of the pg gene in the normal saline group was significantly higher than that in the GLP-1 group (*P* < 0.05; Figure 6(b)). In the anterior intestine, the pg gene expression was significantly higher at 30-120 min than that before injection (*P* < 0.05), and the pg gene expression in the normal saline group was significantly higher than that in the GLP-1 group (*P* < 0.05; Figure 6(c)).

The expression of the gk gene in hepatopancreas showed a trend of continuous increase within 30-120 min after injection, and the gk gene expression in GLP-1 group was significantly higher than that in the normal saline group (*P* < 0.05; Figure 6(d)). The expression of the gk gene in the whole brain showed a trend of firstly decreasing and then increasing after injection. The expression of the gk gene in the GLP-1 group was significantly lower than that in the normal saline group (*P* < 0.05; Figure 6(e)). The expression of the gk gene in the anterior intestine showed a trend of firstly increasing and then decreasing after injection. The expression of the gk gene in the GLP-1 group was significantly higher than that in the normal saline group and before injection (*P* < 0.05; Figure 6(f)).

## 4. Discussion

A number of physiological functions of the incretin hormone GLP-1 in fish have been previously reported, and differences in the functions of GLP-1 in mammals and fish have been identified [26, 27]. The pg gene of *S. denticulatus* was cloned with a full length of 682 bp, encoding 121 amino acids. It was a typical secreted protein without GLP-2 spliceosome. The pg of *S. denticulatus* had the highest homology compared with the cyprinid fishes *S. anshuiensis*, *S. rhinocerous*, and *C. carpio*, up to 96.7%.

Under normal conditions, the pg gene had the highest expression in the anterior intestine, followed by the middle intestine, hepatopancreas, posterior intestine, and whole brain, which might be due to the functional differences of the tissues. GLP-1 could promote glucose absorption, gluconeogenesis, and glycogenolysis in teleost fish [13]. In both fish and mammals, GLP-1 plays a role in the inhibition of feeding in the nerve center [12]. The study by Yuen et al. showed that the expression of the pg gene was highest in the anterior intestine and gallbladder of goldfish, which was similar to the results of our study [28].

In order to further explore the effect of different carbohydrates on the glucose metabolism, we performed an oral administration experiment and found that the blood glucose and hepatic glycogen levels firstly increased and then returned to normal levels. The blood glucose levels of the three groups reached the highest point at 3 h on average, and the blood glucose level of the soluble starch group was significantly higher than that of the other two groups (*P* < 0.05). The hepatic glycogen levels of the glucose group, maltose group, and soluble starch group peaked at 5 h, 5 h, and 3 h, respectively, indicating that polysaccharides were more likely to stimulate an increase in blood glucose level in *S. denticulatus*. Adult *S. denticulatus* tends to feed on algae, aquatic vascular plants, and organic detritus of plants, and the main source of carbohydrates in the food is polysaccharides [29]. The blood glucose and hepatic glycogen levels of the three groups were restored to those before drenching at 12 h. In other carnivorous fish glucose tolerance tests, it was found that the high glycemic load of *O. mykiss* continued for 18 h [30], and the hyperglycemic state of *Acipenser transmontanus* lasted up to 24 h [31], indicating that herbivorous *S. denticulatus* had a stronger glucose metabolism ability than other carnivorous fish. The utilization of carbohydrates in fish varied greatly among different species; the level of digestible sugar in the diet of carnivorous fish generally did not exceed 20%, while that of some omnivorous and herbivorous fish could reach up to 40% [32, 33]. Transformative storage and oxidative decomposition were the main metabolic pathways of carbohydrates in fish. The digested and absorbed carbohydrates were mainly transported to the organs of fish in the form of blood glucose, and then involved the processes of glycolysis, gluconeogenesis, tricarboxylic acid cycle, pentose phosphate pathway, glycogen synthesis, degradation, etc. [34].

The pg gene expression of *S. denticulatus* was significantly affected by drenching with different carbohydrates. At 1 h and 3 h, the pg gene expression of hepatopancreas and anterior intestine in the soluble starch group was significantly higher than that in the other two groups (*P* < 0.05), indicating that polysaccharides and disaccharides stimulated intestinal GLP-1 secretion more than monosaccharides. The hepatopancreas is the main target organ of GLP-1, which increases blood glucose by promoting the gluconeogenesis and glycogenolysis pathway [35]. After drenching, the expression of the pg gene in the whole brain decreased within a short time and slowly returned to the normal level after 5 h, which might be since the whole brain was not the main tissue secreting GLP-1. Many glucose sensors were widely found in the hypothalamus and hindbrain [36], which could respond to the change in blood glucose level and inhibit excessive accumulation of blood glucose by downregulating the expression of pg gene.

In order to further explore the glycemia regulation mediated by GLP-1 in fish, we used the intraperitoneal injection of GLP-1 peptide to analyze the changes in blood glucose levels and pg gene expression. This study determined that the optimal intraperitoneal injection concentration was 1000 *μ*g/kg BW, while the study by Polakof et al. used 100 *μ*g/kg BW GLP-1 for intraperitoneal injection of rainbow trout, which might be based on the differences in feeding habits of experimental fish [10]. The basal blood glucose level of herbivorous fish was lower than that of carnivorous fish, and the rate of recovery from hyperglycemia to basal blood glucose was faster [37]. After intraperitoneal injection of GLP-1, the blood glucose level of *S. denticulatus* showed a trend of first increasing and then decreasing, while the hepatic glycogen content continuously increased, which was similar to that seen in herbivorous grass carp [38] and several carnivorous fish such as rainbow trout [6], copper rockfish [11] and coho salmon (*Oncorhynchus kisutch*) [39]. It was speculated that intraperitoneal and intraventricular injection of GLP-1 peptide increased blood glucose levels in fish with different feeding habits, but the difference in effect time was not clear. In contrast to mammals, the GLP-1 of fish species did not have insulin-like functions. Teleost fishes lost the gene encoding a GLP-1 specific receptor and duplicated the gene encoding a glucagon receptor in the third genome duplication during evolution [26]. The role of GLP-1 in the inhibition of feeding was conserved across species, whereas the role in glucose metabolism has altered during evolution [10, 12] .

Our study found that the effect of intraperitoneal injection of GLP-1 on *S. denticulatus* was rapid with the blood glucose level peaking within 60 minutes, and then decreasing. This result was similar to a study in rats [40], which showed that blood glucose concentrations peaked within 30-60 min after a meal. This might be due to GLP-1, which could enhance the ability of glucose absorption while reducing the oxidation of glucose [41], and there were a large number of GLP-1 receptors in tissues such as the hepatopancreas and intestine [42]. Similar to mammals, there are also many glucose sensors in the central nervous system and peripheral tissues of fish, which can continuously monitor blood glucose changes in the body, trigger hormone secretion, and activate the autonomic nervous system [43].

After injection, it was shown that the expression of the pg gene in the hepatopancreas increased rapidly and then decreased, suggesting that *S. denticulatus* could inhibit the excessive accumulation of blood glucose by reducing the expression of the pg gene. Xie et al. [38] found that the strategy to replace the incretin effect might be to suppress the accumulation of blood glucose by reducing the expression of the pg gene in grass carp. This might be a unique way of blood glucose regulation that was different from carnivorous fish. Similarly, the trend of continuous increase of the expression of the gk gene in hepatopancreas might be due to the blood glucose level increased after intraperitoneal injection of GLP-1; glycolysis in hepatopancreas was promoted. While the expression of pg gene in the whole brain continually decreased after injection. There are glucose receptors in the whole brain that continuously monitor blood glucose changes and may make rapid negative feedback adjustments when blood glucose level rises [44]. The central system in the brain has detected the elevation of blood glucose and hepatic glycogen levels, and it could control the blood glucose and hepatic glycogen to return to the normal levels by downregulating the expression of the gk gene. We noticed that the expression level of the pg gene in the hepatopancreas decreased rapidly, while the level of the pg gene expression in the anterior intestine remained high. This might be related to the stronger ability of the hepatopancreas to metabolize GLP-1, and GLP-1 was mainly secreted by intestinal L cells. After intraperitoneal injection of GLP-1, the intestine would be affected by the false appearance of eating, which led to the increase of blood glucose and the expression of pg and gk genes in a short time [45].

Interestingly, the injection of normal saline resulted in a high level of pg gene expression in the hepatopancreas and anterior intestine for 2 h. The injected normal saline was absorbed into the blood circulation through the abundant capillaries, resulting in a decrease in blood glucose concentration detected by the hypothalamus. In response to hypoglycemia, the body induced the upregulation of pg gene expression, and more GLP-1 was secreted to regulate blood glucose homeostasis. This process might involve the role of the hypothalamus-pituitary-interrenal (HPI) axis, which was analogous to the hypothalamus-pituitary-adrenal (HPA) axis in mammals. It has been confirmed that the neurohumoral regulatory response induced by hypoglycemia included the termination of the release of endogenous insulin, the release of glucagon, and the activation of the pituitary-adrenal axis [46, 47]. Hypoglycemia could activate GLP-1 neurons, acting on GLP-1 receptors, and then further activated corticotropin-releasing hormone- (CRH-) producing neurons in the paraventricular nucleus, triggering HPA axis responses in rats [48]. It was, thus, inferred that the HPI axis might be involved in the regulation of pg expression and GLP-1 secretion in *S. denticulatus*.

Taken together, the complete sequence of the pg gene in *S. denticulatus* was cloned, and the GLP-1 fragment was found. Polysaccharide was more effective than monosaccharide in stimulating the blood glucose levels of *S. denticulatus*. We further verified the crucial glycemic effect of GLP-1 by intraperitoneal injection. *S. denticulatus* inhibited the excessive accumulation of blood glucose by reducing the expression of the pg gene, which might be one of the reasons why herbivorous fish were more tolerant to high levels of dietary carbohydrates than carnivorous fish. The effect of intraperitoneal injection of GLP-1 on the expression of pg andgk gene in hepatopancreas, whole brain, and anterior intestine was time efficient, and it was speculated that GLP-1 in *S. denticulatus* might have a similar “gut-brain-liver” pathway to mammals, although further research was needed for confirmation [40]. This study could provide a novel perspective for further understanding the process of glycemia regulation of herbivorous fish mediated by GLP-1.

## Data Availability

The gene complete sequence, gene expression, primer sequence, and phylogenetic analysis data used to support the findings of this study are included within the original article and supplementary information file.

## Additional Points

*Highlights*. (i) The hyperglycemic effect of GLP-1 was essentially consistent across many fish species with different feeding habits, in contrast to mammals. (ii) In *Spinibarbus denticulatus*, polysaccharide was more effective than oligosaccharide in stimulating both an increase in blood glucose and intestinal GLP-1 secretion. (iii) In *S. denticulatus*, excessive accumulation of blood glucose was inhibited by the downregulation of proglucagon (pg) gene expression, which might be a particular pathway for glycemia regulation in herbivorous fish.

## Supplementary Materials

Supplementary Materials All the supplementary tables and figures are included in the file. Supplementary Table 1: the primers used for pg gene cloning. Supplementary Table 2: the primers used for real-time PCR. Supplementary Table 3: pg amino acid identity of *S. denticulatus* and other vertebrates. Supplementary Table 4: the basal glucose blood value of *S. denticulatus*. Supplementary Figure 1: agarose electrophoresis figure of total RNA. Supplementary Figure 2: schematic representation of *S. denticulatus* proglucagon genes and peptide products predicted for the endocrine pancreas. Supplementary Figure 3: the 3D model of *S. denticulatus* pg protein. Supplementary Figure 4: comparison of pg amino acid sequences of *S. denticulatus* and other vertebrates. Supplementary Figure 5: phylogenetic tree of pg amino acid sequences of *S. denticulatus* and other vertebrates.
