# Supplementary material for: Study on the Hyperglycemic Effect of GLP-1 in Spinibarbus denticulatus by Oral Administration and Intraperitoneal Injection Methods
Source: Aquac Nutr. 2023 Apr 3;2023:9969406. doi: 10.1155/2023/9969406 (PMC10085660; doi:10.1155/2023/9969406)
Supplement: Supplementary Materials — All the supplementary tables and figures are included in the file. Supplementary Table 1: the primers used for pg gene cloning. Supplementary Table 2: the primers used for real-time PCR. Supplementary Table 3: pg amino acid identity of S. denticulatus and other vertebrates. Supplementary Table 4: the basal glucose blood value of S. denticulatus. Supplementary Figure 1: agarose electrophoresis figure of total RNA. Supplementary Figure 2: schematic representation of S. denticulatus proglucagon genes and peptide products predicted for the endocrine pancreas. Supplementary Figure 3: the 3D model of S. denticulatus pg protein. Supplementary Figure 4: comparison of pg amino acid sequences of S. denticulatus and other vertebrates. Supplementary Figure 5: phylogenetic tree of pg amino acid sequences of S. denticulatus and other vertebrates. [file 9969406.f1.docx]

**Supplementary Materials**

**Table S1** The primers used for *pg* gene cloning

| Primers | Sequences (5’→3’) | Purpose |
| --- | --- | --- |
| *pg*-1F | TTCTTCTGCTCCTCTTTG | Cloning of the core sequence |
| *pg*-1R | TTGGACAGGGTGCGTTAG |  |
| *pg*-2F | TCAACTCTTCCATCCATTGAAC |  |
| *pg*-2R | TGGACAGGGTGCGTTAGA |  |
| *pg*-gsp1 | CCTGGTCTCCAAGTATTTGCTGTAGTCG | *pg* gene 5 'end amplification |
| *pg*-gsp2-L | GCAGGACCAGGCGGCCCAGAACTTTGTG | *pg* gene 3 'end amplification |
| *pg*-gsp2-S | AGACCGAGTGTGCGAGAGAGAAAACTGG |  |
| UPM-long | CTAATACGACTCACTATAGGGCAAGCAGTGGTATCAACGCAGAGT | *pg* gene 5'、3' end amplification(the universal primer) |
| UPM-short | CTAATACGACTCACTATAGGGC |  |

Note: gsp1: downstream primer; gsp2: upstream primer; UPM: the universal primer in the kit.

**Table S2** The primers used for real-time PCR

| Primers | Sequences (5’→3’) |
| --- | --- |
| *pg*-yg-F | AGAACTTTGTGGCCTGGCTAA |
| *pg*-yg-R  *gk*-yg-F  *gk*-yg-R | CTCGCACACTCGGTCTTTTTC  TGCGTCTTGTGCTGCTAA  TGCTTTCTGTCCCCTGTG |
| β-ActinF1 | ACGGTATTGTGACCAACTGG |
| β-ActinR1 | TGTGGGTCACACCATCACC |
| EF- 1α-yg-F | ACTCCACCGAGCCCCCCTAC |
| EF- 1α-yg-R | TGTCCCCATGCCATCCAGAA |
| EF- 1αF1 | TCAAGTATGCCTGGGTGT |
| EF- 1αR1 | GCAATGTGAGCAGTGTGG |

**Table S3** *pg* amino acid identity of *S. denticulatus* and other vertebrates

| Species | Homology (%) | | GenBank ID |
| --- | --- | --- | --- |
| *Sinocyclocheilus* *anshuiensis* | 96.7 | | XP_016309506.1 |
| *Sinocyclocheilus* *rhinocerous* | 96.7 | XP_016428443.1 | |
| *Cyprinus* *carpio* | 95.0 | XP_018921072.1 | |
| *Schizothorax* *prenanti* | 95.0 | AKC03099.1 | |
| *Carassius* *auratus* | 92.6 | AAB39563.1 | |
| *Ictalurus* *punctatus* | 66.1 | AAS57650.1 | |
| *Danio* *rerio* | 62.0 | NP_001229699.1 | |
| *Oncorhynchus* *mykiss* | 64.5 | AAB34505.1 | |
| *Sebastes* *caurinus* | 62.0 | AAS57658.1 | |
| *Hoplobatrachus* *rugulosus* | 48.7 | AAL35758.1 | |
| *Callorhinchus* *milii* | 53.7 | AFP03248.1 | |
| *Heloderma* *suspectum* | 51.7 | AAB51129.1 | |
| *Sus* *scrofa* | 52.5 | AAO88211.1 | |
| *Gallus* *gallus* | 53.3 | AAB34506.1 | |
| *Mus* *musculus* | 52.5 | CAA86902.1 | |
| *Xenopus* *laevis* | 47.5 | AAB65661.1 | |

**Table S4** The basal glucose blood value of *S. denticulatus*

| Time | Samples (n) | Blood glucose  (mmol/L) | | 95% confidence interval  Means ± SD  Means ± S.D.  Minimum Maximum |
| --- | --- | --- | --- | --- |
| After fasting for 48 h | 9 | 3.35  3.27  4.30  3.49  4.41  4.29  4.68  4.78  5.07  3.27  3.25  3.09  3.83  3.48  4.28  3.81  3.22  5.34 |  | 4.07 ± 0.66 3.46 4.68 |
| After fasting for 288 h | 9 |  |  | 3.73 ± 0.67 3.24 4.23 |


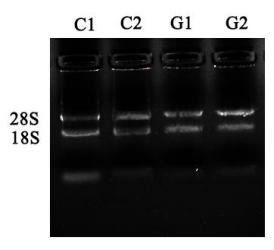


**Fig. S1.** Agarose electrophoresis figure of total RNA. C1, C2 indicates two midgut samples; G1, G2 indicates two hepatopancreas samples.


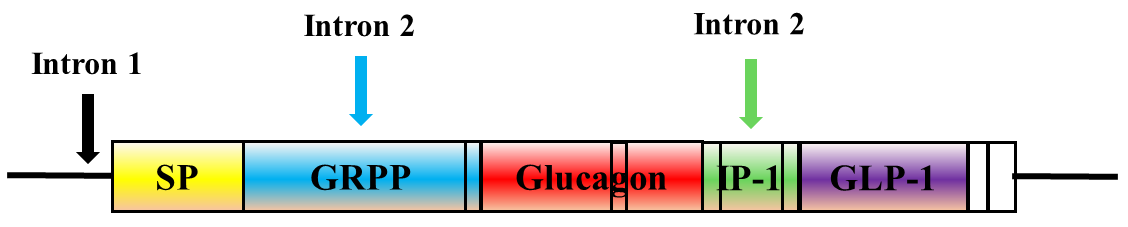


**Fig. S2.** Schematic representation of *S. denticulatus* proglucagon genes and peptide products predicted for endocrine pancreas. Vertical lines in gene structures indicate Putative proteolytic cleavage sites. SP: signal peptide; GRPP: glicentin-related polypeptide; IP: intervening peptide.


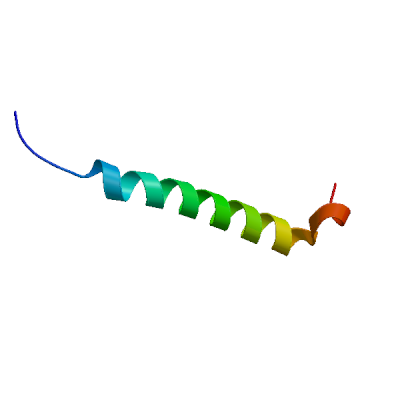


**Fig. S3.** The 3D model of *S. denticulatus* *pg* protein, a typical 3D spatial conformation of a secreted protein.


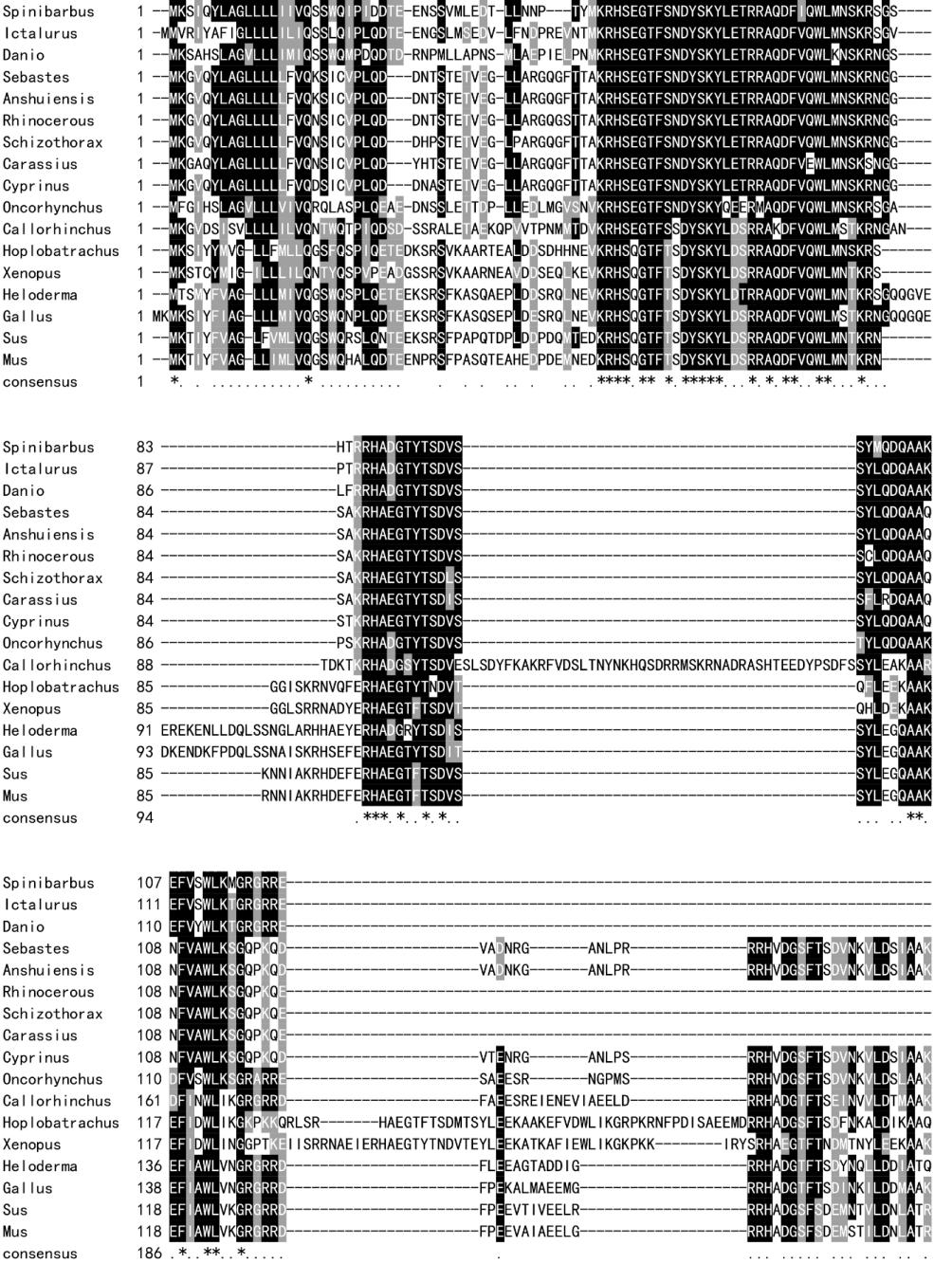


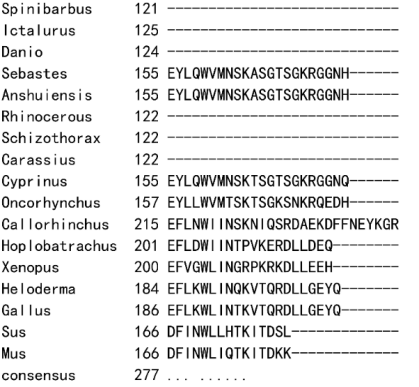


**Fig. S4.** Comparison of *pg* amino acid sequences. The colored amino acids highlight the differences in conservation of the amino acids between species. Asterisks mark amino acid residues homologous between all these PGs. Species names and GenBank accession numbers used in the alignment were same with the Table S3.


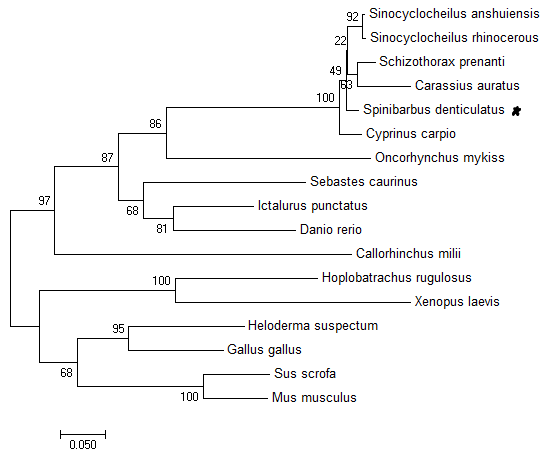


**Fig. S5.** Phylogenetic tree of *pg* amino acid sequences of *S. denticulatus* and other vertebrates
